# Directly converted iNeuron as a screening model for pathogenic variants

**DOI:** 10.18632/oncotarget.14111

**Published:** 2016-12-22

**Authors:** Su Min Lim, Chang-Seok Ki, Seung Hyun Kim

**Affiliations:** Department of Neurology, College of Medicine, Hanyang University, Seoul, Republic of Korea and Cell Therapy Center, Hanyang University Hospital, Seoul, Republic of Korea

**Keywords:** direct conversion, induced neuron, neurodegenerative disease, patient-specific cell model

Recent advances in genetic technologies have undoubtedly contributed to identify a flood of rare variants including novel or pathogenic variants. However, one remarkable finding of the Exome Aggregation Consortium (ExAC) is that many likely benign variants have been misclassified as harmful [1]. Therefore, reliable cell modeling systems are crucible to hunt for evidence that newly identified variant has a functional role in disease before declaring that it is pathogenic. Moreover, these models should at least recapitulate human pathological findings.

Cell death mechanisms of diverse neurodegenerative diseases including Alzheimer’s disease, Parkinson’s disease and Amyotrophic Lateral Sclerosis (ALS) have been poorly understood due to the lack of clinically relevant models that recapitulate the specific molecular pathogenesis of a disease. Limited information obtainable from postmortem neural tissue and animal model fails to establish faithful analogs to understand ongoing cell death mechanism of neurodegenerative disease. Hence, modeling pathophysiological cascades with human adult somatic cells carrying pathogenic variants provides fascinating prospects.

Adult somatic cells with neurodegenerative disease including patient skin fibroblasts or reprogramming of fibroblasts to induced pluripotent stem cells (iPSC), which are capable of differentiating to neurons have been developed as models to provide better understanding of the disease pathology [2, 3]. However, pathological neuronal features are not present in patient fibroblasts whereas the generation of iPSC-derived neurons requires intricate procedures and has low reprogramming efficiency [4]. Moreover, developmental, environmental, and age-related alterations in epigenetic events are lost upon establishing iPSCs, which are limitations associated with the use of iPSC-derived neurons [5]. The recent promising approach to transdifferentiate terminally differentiated cells into another cell fate does not require complex culture procedures: Direct conversion of fibroblast into induced neuron (iNeuron). Compared to iPSC, iNeuron is simple, rapid, and within higher reprogramming efficiency, which fast-tracks the reprogramming process (Figure 1). The down-regulation of a single RNA binding polypyrimidine-tract-binding protein 1 (PTBP1) in fibroblast to establish iNeuron and sort out the untransduced cells with puromycin has allowed highly efficient cell modeling for neurodegenerative diseases [6–8]. Moreover, assuming that the patient fibroblast and its directly converted iNeurons have the same developmental age, the epigenetic alterations might be retained in iNeurons, which is why iNeuron is an efficient model to study the pathogenesis of neurodegenerative disease.

**Figure 1:**
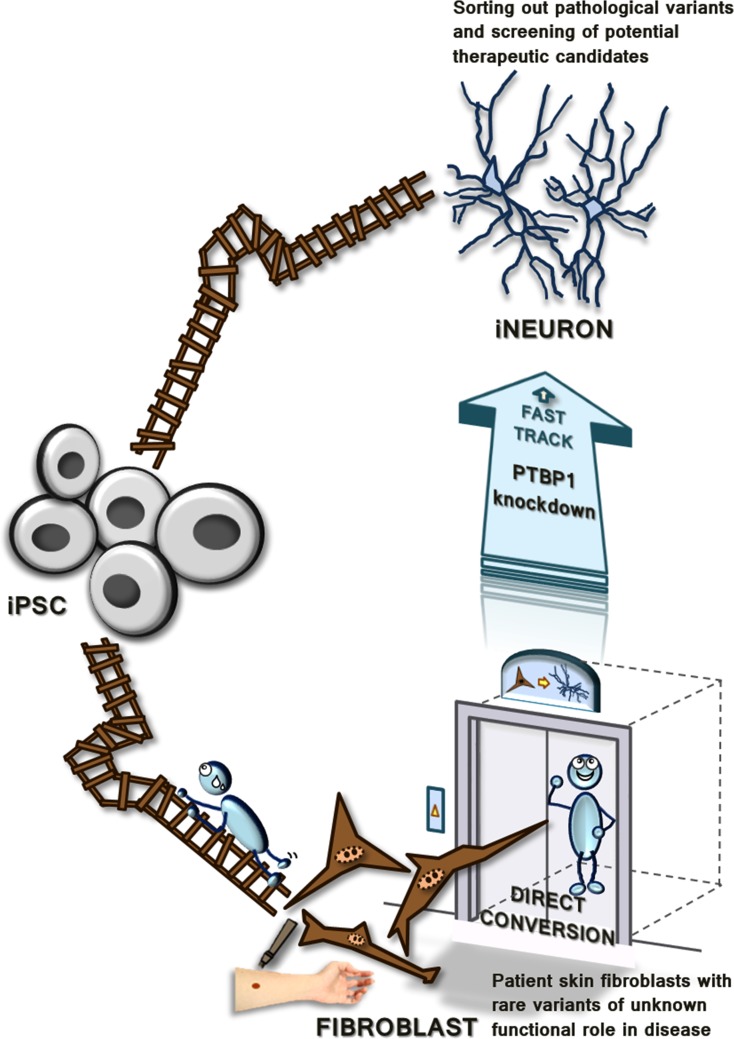
Direct conversion from human skin fibroblasts to iNeurons to fast-track the reprogramming process

In our recent study on ALS, we cultured skin fibroblasts from ALS patients carrying pathogenic variants in the *fused in sarcoma* (*FUS*) gene, reprogrammed, and grew into mature neurons [7]. In our experiment, iNeuron models from patients with mutant FUS at the C-terminal nuclear localization signal (NLS) region of the protein had aberrant cytosolic mislocalization with nuclear clearance. Such pathological features of mutant FUS, which recapitulate the pathological phenotype of the autopsied ALS patient, were specific for patient-derived iNeurons as they do not occur in the patient’s fibroblasts. Our study therefore revealed that iNeurons may provide a reliable model to estimate each patient-specific pathology in ALS-FUS.

We used iNeuron models to illuminate the disease pathology in another devastative neurodegenerative disease. In this study, we not only compared iNeurons from Krabbe disease (KD) patients to disease-free healthy control iNeurons, but also added a potent disease-causing factor psychosine to the control iNeurons to establish the causal role of its accumulation in KD [8]. KD is an autosomal recessive neurodegenerative disorder caused by lysosomal enzyme ß-galactosylceramidase (GALC) deficiency. We reported that adult-onset KD iNeurons showed diminished GALC activity and increased levels of psychosine, the toxic substrate that accumulates in KD. Neurite fragmentation along with abnormal lysosomal or mitochondrial function was observed, suggesting autonomous neuronal toxicity in KD pathology. Increase of psychosine levels in healthy iNeurons was sufficient to induce pathology found in the patient iNeurons. This provides compelling evidence for the causal role of neurotoxic psychosine accumulation in KD and highlights autonomous neuronal dysfunction in adult-onset KD.

Therefore, use of reliable screening system for sorting out which variants currently considered pathogenic to be actually benign is absolutely important, and *vice versa* as well. For example, patient-specific iNeuron model provide FUS (p.Q519E) variant considered as benign in non-neuronal cell models to be turned out as pathogenic depending on the pathological features exhibited in more disease-relevant models. Therefore, the variants that were considered benign in less disease-related cell models should be revisited to estimate their pathogenicity in more disease-relevant models.

In order to fully elucidate which variants are actually causative for disease will be a challenging task. Before declaring that a variant is pathogenic or non-pathogenic, researchers should hunt for evidence that the variant does or does not provide mechanistic insights into the functional link between the genetic variant and the disease. More rigorous evaluation using multiple approaches to establish the most adequate model to find the genetic causes of disease is therefore needed. Using patient-specific iNeurons, future studies should focus on mediating gene editing in patient iNeurons to correct underlying pathogenic variants and ameliorate disease features or increase cellular survival. This must be done not only to reveal the disease causality but also to help develop precise and personalized medicine for the treatment of a specific genetic defect. Diseases should be dissected at a small scale as well as a large scale to address biological questions in neurodegenerative diseases. High-throughput single neuron analysis and time-series single cell measurements can compare neuron-neuron transcriptional variability to understand cellular heterogeneity in neurological disease. Multicellular culture systems such as the coculture of the supernatant derived from healthy neurons with disease-derived glial cells or multicellular organoids of healthy glial cells with disease-derived neurons will be an efficient path to address long-standing questions regarding multicellular pathogenesis in neurodegenerative diseases. Collectively, iNeurons may be a reliable model for investigating and understanding the genetic causes of neurodegenerative diseases.

## References

[1] Lek M (2016). Nature.

[2] Cotan D (2011). FASEB J.

[3] Devlin AC (2015). Nat Commun.

[4] Soldner F (2009). Cell.

[5] Kyttala A (2016). Stem Cell Reports.

[6] Xue Y (2013). Cell.

[7] Lim SM (2016). Mol Neurodegener.

[8] Lim SM (2016). Oncotarget.

